# Multi-level predictors of psychological problems among methadone maintenance treatment patients in difference types of settings in Vietnam

**DOI:** 10.1186/s13011-019-0223-4

**Published:** 2019-09-18

**Authors:** Tuan Anh Le, Mai Quynh Thi Le, Anh Duc Dang, Anh Kim Dang, Cuong Tat Nguyen, Hai Quang Pham, Giang Thu Vu, Chi Linh Hoang, Tung Thanh Tran, Quan-Hoang Vuong, Tung Hoang Tran, Bach Xuan Tran, Carl A. Latkin, Cyrus S. H. Ho, Roger C. M. Ho

**Affiliations:** 1 National Institute of Hygiene and Epidemiology, Hanoi, 100000 Vietnam; 2 Institute for Preventive Medicine and Public Health, Hanoi Medical University, Hanoi, 100000 Vietnam; 3 Institute for Global Health Innovations, Duy Tan University, Postal address: No. 73 Hoang Cau street, Hanoi, Da Nang, Vietnam; 4 Center of Excellence in Evidence-based Medicine, Nguyen Tat Thanh University, Ho Chi Minh City, 700000 Vietnam; 5 Center of Excellence in Behavior Medicine, Nguyen Tat Thanh University, Ho Chi Minh, 700000 Vietnam; 6 Centre for Interdisciplinary Social Research, Phenikaa University, Yen Nghia, Ha Dong, Hanoi, 100803 Vietnam; 7 Faculty of Economics and Finance, Phenikaa University, Yen Nghia, Ha Dong, Hanoi, 100803 Vietnam; 8 Institute of Orthopaedic and Trauma Surgery, Vietnam – Germany Hospital, Hanoi, 100000 Vietnam; 9 Bloomberg School of Public Health, Johns Hopkins University, Baltimore, MD 21205 USA; 10 Department of Psychological Medicine, National University Hospital, Singapore, 119074 Singapore; 11 Department of Psychological Medicine, Yong Loo Lin School of Medicine, National University of Singapore, Singapore, 117599 Singapore; 12 Biomedical Global Institute of Healthcare Research & Technology (BIGHEART), National University of Singapore, Singapore, 117599 Singapore

**Keywords:** Psychological, Depression, Anxiety, Stress, Methadone, Vietnam

## Abstract

**Background:**

Methadone, a long-acting opioid agonist maintenance treatment (MMT) is used to treat opioid addiction by preventing opioid withdrawal and reducing cravings. However, it is important to note that mental conditions may persist, or even remain undetected while methadone maintenance treatment is ongoing. This study aimed to examine the level of psychological problems among MMT patients at public and private health facilities and identify associated factors.

**Method:**

From January to September 2018, a cross-sectional study was performed in Nam Dinh province, one of the largest epicenters providing HIV/AIDS surveillance and treatment services in the North of Vietnam. 395 male respondents currently receiving MMT agreed to participate in a face-to-face interview. Depression, Anxiety and Stress Scale-21 (DASS-21) were used to assess psychological problems among patients.

**Results:**

The percentage of patients suffering from mild to extremely severe anxiety was the highest among psychological problems (18%). 2.8% of participants had mild depressive symptoms and the percentage of those having mild or moderate stress was approximately 4%. In addition, the longer treatment duration, the lower mental health scores regarding three types of psychological problems. Respondents who received MMT services in public health facilities were more likely to have a higher score of all psychological problems. Participants who lived with partners or spouse, having higher monthly family income had a lower likelihood of having severe depression and stress status. Freelancers or blue-collars/farmers had lower score of depression and anxiety compared to people being unemployed.

**Conclusion:**

This study suggests that among our sample, MMT patients receiving treatment in public health facilities might have higher rate of psychological problems, including depression, anxiety, and stress than that of those in the private health facility. These results highlight the necessity of taking psychological counseling adequately for MMT patients and psychological assessment should be prioritized in the early stage of treatment.

## Background

Methadone Maintenance Treatment (MMT) is a long-term or permanent treatment, which replaces opioids by other substitutes to mitigate physical and psychosocial hardships for patients [1]. Psychiatric disorders have been cited as a significant barrier for individuals to optimally adhere to opioid-dependent treatments. Drug users suffering from psychological health issues have a higher risk of having lower quality of life [2, 3], suicide attempts [4, 5] and an even higher rate of mortality [6]. Mental health problems can be considered as driving force in diminishing MMT treatment outcomes such as higher rate of involving in HIV-related risk behaviors [7], interfering with therapeutic compliance with MMT and increasing retention in methadone treatment [8]. Thus, identifying and giving treatments of psychiatric co-morbidities for opioid-addicted patients is crucial to enhance the efficacy of MMT programs.

Evidence in the literature shows that there is a considerable prevalence of MMT patients experiencing psychological problems in which depression and anxiety are overwhelming disorders [9, 10]. A study conducted in China revealed that 57.5% of MMT users reported to suffer from depressive symptoms, and more than one in four (25.8%) had suicidal ideation [11]. In term of depression, a study of Weissman et al. showed that approximately one-third of MMT patients in the community had moderately to severely depression [12], 50% of MMT patients were found to experience depression based on study of Peles et al. [13], while the rate of lifetime depression prevalence among opioid dependence patients engaging in treatment programs ranged from 20 to 50% [14].

Notably, the satisfaction of medical services is also considered as a factor related to the mental health status of patients [15]. In order to meet the demand of patients and increase the accessibility of healthcare services, private health facilities also provide MMT treatment for drug users. Public health facility is referred to have a better quality of treatment including licensed and certified medical staff [16], higher diagnostic accuracy [17], better adherence to medical management standards [18] and higher rates of treatment success [19]. On the other hand, private health facility has succeeded in providing a better quality of services, for example, reducing the waiting time [20], improving hospitality from providers [21] and lowering the proportion of patients experiencing stigma and discrimination [22].

The expansion of the MMT program in Vietnam has increased rapidly in recent years. Since the first MMT clinic introduced in 2008, the government of Vietnam has made great commitment to expanding the MMT program to cover a large number of drug users nationwide [23, 24]. The Ministry of Health in Vietnam also emphasizes the need for psychological healthcare when receiving MMT [25]. Prior studies have assessed the prevalence of psychiatric problems among MMT patients, ranged from 26.8 to 43.1%. [6, 22]. However, there is little evidence taking account of the impact of different types of MMT outpatient clinics. Therefore, this study aimed to examine the level of psychological problems among MMT patients at public and private health facilities and other factors contributing to a higher risk of suffering from these psychological problems.

## Methods

### Study setting and subjects

We conducted a cross-sectional study from January 2018 to September 2018 in Nam Dinh province. Nam Dinh is one of the largest epicenters offering MMT services in the North of Vietnam. The study settings took place in three MMT clinics (Giao Thuy district health center, Dai Dong private health facility and Giao Thuy center for social evils prevention). We selected the clinics based on two eligibility criteria [1] providing methadone treatment services following the official guidelines of the Ministry of Health in Vietnam and [2] the period of offering Methadone treatment services was at least 12 months.

We used convenience sampling technique to recruit participants. Participants were chosen based on four eligibility criteria, which were [1] being at least 18 years old; [2] undergoing MMT services of settings mentioned above; [3] agreeing to participate in the study and [4] being able to answer questions from data collectors. A total of 395 respondents agreed to take part in the study. The percentage of patients in each health facility was 49.4% (Dai Dong private health facility), 25.3% (Giao Thuy district health center) and 25.3% (Giao Thuy center for social evils prevention).

### Measure and instruments

20-min face-to-face interviews were carried out to collect data. The interviewers were well-trained researchers. Medical staffs in the clinics were not invited to participate in data collection in order to avoid social desirability bias. Participants were asked to involve in the study when they attended these clinics for treatment or counseling services. Eligible respondents were identified based on the feedback from medical staffs. To secure participants’ confidentiality, the interviews occurred in a small private counseling room. Participants could take part in the study after being introduced the study objectives, benefits, drawbacks and provided verbal informed consent.

A pilot survey was conducted prior to the main study among 40 respondents with different social characteristics, including ages, employment and educational level to test and refine the questionnaire. Minor changes regarding wording were made based on the feedback of participants. A structured questionnaire was applied to the following information:

#### Socioeconomic characteristics

Participants self-reported general information, including age, marital status, occupation, educational level and monthly income.

#### Mental health status

In order to assess the mental health status among participants, we used the Depression, Anxiety, and Stress Scale-21 (DASS-21). This tool consists of 21 items, which measure three sub-scales of emotional states, including depression, anxiety, and stress. Each sub-scale contains 7 questions and the answer for each question ranges from 0 (Did not apply to me at all) to 3 (Applied to me very much, or most of the time). Participants were asked to indicate the presence of a symptom over the past week. Scores for three emotional states were calculated by summing the points for the relevant items (question 3, 5, 10, 13, 16, 17, 21 for depression; question 1, 6, 8, 11, 12, 14, 18 for stress; question 2, 4, 7, 9, 15, 19, 20 for anxiety) and double up. There were 5 levels for the cut-off point based on DASS-21 scoring containing: normal, mild, moderate, severe, extremely severe. The DASS can be a useful assessment of disturbance, either the level of severity of patients’ symptoms or how the patient’s response to treatment [26].

#### Health risk behavior

Participants were asked about whether they currently drink alcohol, smoke tobacco or use drugs. In term of quality of life, “How your quality of life change between before and after having MMT service?” was also mentioned in the questionnaire. Participants also reported their HIV-infection status.

#### Methadone maintenance treatment-related- characteristics

Participants self-reported their overall assessment regarding the quality of MMT service in the health facility where they attended and their satisfaction for traveling to MMT facility to take pills. Level of adherence to MMT was self-assessed using a Likert scale, including 5 options from “Very good” to “Very bad”. Moreover, a 100-point visual analog scale (VAS) was also employed to detect patients’ adherence, with a score range from 0 “incompletely adherence” to 100 “completely adherence”. The threshold for optimal adherence was 95%.

#### Social/family support

To identify the support from social and family, we asked participants about whether they received the support during MMT duration.

### Statistical analysis

STATA version 12 (Stata Corp. LP, College Station, United States of America) were used to analyze data. A Chi-square test, a Mann Whitney test and a Kruskal-Wallis test were used for analyzing demographic characteristics of participants as well as health risk behaviors, depression situation. Multivariate Tobit regression was applied to examine factors associated with a psychological problem. The Tobit regression model is designed to assess linear relationships between variables when dependent variables censored from below and above [27]. In this study, each of the psychological subscales may range between 0 and 42. To identify the reduced regression model, we applied a forward stepwise selection strategy with the threshold of less than 0.2. A *p*-value < 0.05 was considered as statistical significance.

### Ethics approval

Ethics approval was reviewed and granted by the Institutional Review Board of National Institute of Hygiene and Epidemiology.

## Results

Table 1 presents the socioeconomic characteristics of participants. The percentage of participants from 30 to 40 years old was highest (42.8%). A high proportion of participants had secondary school education (60.0%), lived with partners or spouse (77.0%) and were freelancers (35.2%). Prevalence of smoking was found to be high in the study areas (81%) and more than half of participants (53.4%) reported that they consume alcohol. Approximately two-thirds of participants had a history of injecting drug (63.8%) and only 5% of participants were concurrently using drug.

**Table 1:** Socio-economic characteristics of respondents

Characteristics	Private facility		State facility		Total		*P*-value
	*n*	%	*n*	%	*n*	%	
Total	195	49.4	200	50.6	395	100	
Age group							
Under 30	29	14.9	19	9.5	48	12.2	0.03*
30–40	89	45.6	80	40.0	169	42.8	
41–50	60	30.8	66	33.0	126	31.9	
Above 50	17	8.7	35	17.5	52	13.2	
Education							
Less than secondary	33	16.9	33	16.5	66	16.7	0.94*
Secondary school	118	60.5	119	59.5	237	60	
More than secondary	44	22.6	48	24	92	23.3	
Marital status							
Single	38	19.5	29	14.5	67	17	0.07*
Live with partners/spouse	150	76.9	154	77	304	77	
Divorced/widow	7	3.6	17	8.5	24	6.1	
Occupation							
Unemployment	13	6.7	20	10	33	8.4	0.06*
Freelancer	63	32.3	76	38	139	35.2	
Blue collar/farmer	45	23.1	47	23.5	92	23.3	
Business	11	5.6	17	8.5	28	7.1	
Others	63	32.3	40	20	103	26.1	
Quintile average family income							
Quintile 1	39	20	41	20.5	80	20.3	0.89*
Quintile 2	37	19	45	22.5	82	20.8	
Quintile 3	42	21.5	39	19.5	81	20.5	
Quintile 4	43	22.1	39	19.5	82	20.8	
Quintile 5	34	17.4	36	18	70	17.7	
Ever injected drugs	121	62.1	131	65.5	252	63.8	0.48*
Alcohol drink	114	58.5	97	48.5	211	53.4	0.05*
Smoke	163	83.6	157	78.5	320	81	0.20*
Concurrent drug use	15	7.7	8	4	23	5.8	0.12*
	Median	IQR	Median	IQR	Median	IQR	
Age	38	33–44	41	34–48	39	33–46	0.01^#^
Monthly family income (USD)	344	215–430	301	215–430	344	215–430	0.50^*#*^
Age of onset of drug use	25	20–30	25	21–31	25	20–31	0.05^*#*^
MMT duration (years)	2	1–5	3	2–6	3	1–5	0.02^*#*^

*Chi square test, ^#^Mann-Whitney rank sum test

According to Table 2, almost all of participants reported that their life changed better after using MMT (95.4%). Only 3.7% were HIV-infected. 43.3% of participants reported optimal MMT adherence and about 77% of responders received support from family during MMT treatment.

**Table 2:** MMT – related characteristic of participants

Characteristics	Private facility		State facility		Total		*P*-value
	*n*	%	*n*	%	*n*	%	
Quality of life change after using MMT							
Better	185	94.9	192	96.0	377	95.4	0.59
Unchanged	10	5.1	8	4.0	18	4.6	
HIV test results							
Negative	178	92.7	169	89.9	347	91.3	0.47
Positive	7	3.7	7	3.7	14	3.7	
Unknown	7	3.7	12	6.4	19	5.0	
MMT adherence VAS							
Optimal adherence	96	49.2	75	37.5	171	43.3	0.02
Suboptimal adherence	99	50.8	125	62.5	224	56.7	
Receiving support for MMT							
Health workers at MMT facility	97	49.7	66	33.0	163	41.3	< 0.01
Relatives in family	167	85.6	140	70.0	307	77.7	< 0.01
Peer in MMT	41	21.0	26	13.0	67	17.0	0.03
Neighbors/other acquaintances	9	4.6	7	3.5	16	4.1	0.57

Table 3 highlights the psychological characteristics of participants according to different types of health facilities. Regarding depression, only 2.8% of participants had mild depressive symptoms and about 1% of participants suffered from moderate, severe and extremely severe depression. About 8% of respondents underwent mild (8.6%) or moderate anxiety (8.1%). The percentage of those having mild or moderate stress was approximately 4%. The mean score of all psychological dimension among participants using services in MMT public facilities was significantly higher than those who use private MMT service. These differences were statistically significant (*p* < 0.05).

**Table 3:** Psychological problems among MMT patients

Characteristics	Private facility		Public facility		Total		*P*-value
	*n*	%	*n*	%	*n*	%	
Total	195	49.4	200	50.6	395	100.0	
Depression							
Normal	187	95.9	193	96.5	380	96.2	0.71*
Mild	6	3.1	5	2.5	11	2.8	
Moderate	1	0.5	1	0.5	2	0.5	
Severe	0	0.0	1	0.5	1	0.3	
Extremely severe	1	0.5	0	0.0	1	0.3	
Anxiety							
Normal	170	87.2	154	77.0	324	82.0	0.05*
Mild	13	6.7	21	10.5	34	8.6	
Moderate	9	4.6	23	11.5	32	8.1	
Severe	2	1.0	2	1.0	4	1.0	
Extremely severe	1	0.5	0	0.0	1	0.3	
Stress							
Normal	188	96.4	191	95.5	379	96.0	0.88*
Mild	6	3.1	8	4.0	14	3.5	
Moderate	1	0.5	1	0.5	2	0.5	
	Mean	SD	Mean	SD	Mean	SD	
DASS-21 sub-scale score							
Depression	1.2	3.2	1.7	3.0	1.5	3.1	< 0.01^#^
Anxiety	2.9	3.9	4.2	3.8	3.6	3.9	< 0.01^#^
Stress	1.7	3.4	2.5	3.6	2.1	3.5	< 0.01^#^

*Chi square test, ^#^Mann-Whitney rank sum test

**Fig. 1:**
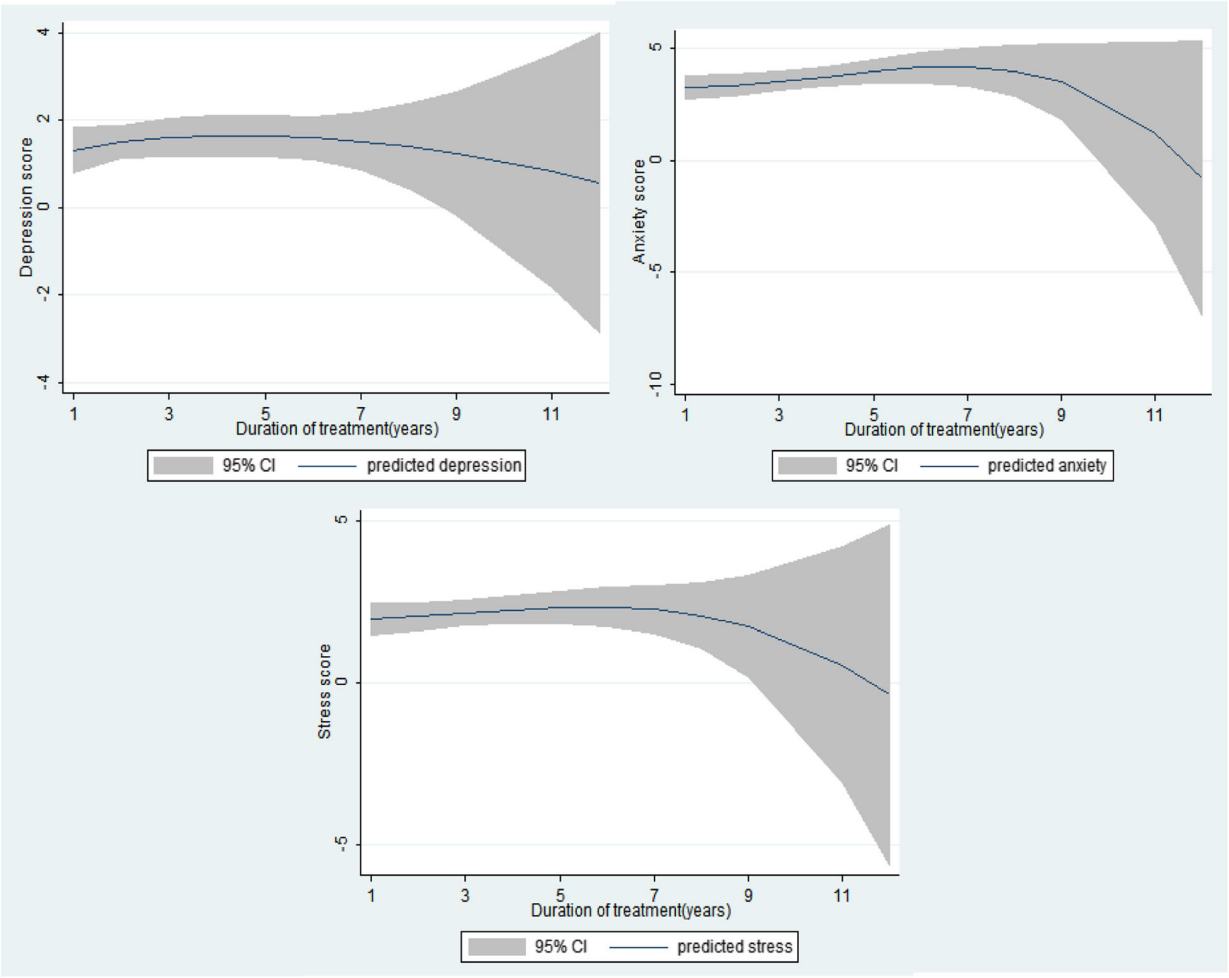
Mean DASS 21 Depression score for duration of MMT. Abbreviation: DASS 21 - the Depression, Anxiety, and Stress Scales

Figure 1 indicated that the longer of treatment duration, the lower mental health scores regarding three types of psychological problems. Anxiety score and stress score a significant decrease during the time of treatment.

The results of the regression models were displayed in Table 4. Respondents who received MMT services in public health facilities were more likely to have a higher score of all psychological problems. Participants who living with partners or spouse had a lower likelihood of having more severe depression and stress status than those who are single. People having higher monthly family income and health workers at MMT facility as supporters have a negative association with DASS-21 score. Compared to being unemployed, freelancer or blue-collar/farmer were associated with a lower score of depression and anxiety. By contrast, having the unchanged quality of life after using treatment had a positive association with anxiety and stress sub-scale score.

**Table 4:** Factors associated with mental health of MMT patients

Characteristics	Depression		Anxiety		Stress	
	Coef.	95% CI	Coef.	95% CI	Coef.	95% CI
Marital status (vs Single)						
Live with partners/spouse	−3.67*	−5.75; − 1.60			−2.62*	−4.27; − 0.97
Divorced/widow	− 3.15	−6.81; 0.50				
Occupation (vs Unemployment)						
Freelancer	−2.33*	−4.24; − 0.41	−2.00*	−3.35; −0.64		
Blue collar/farmer			−1.73*	−3.25; − 0.21		
Business					2.74	−0.12; 5.59
Others	1.94	−0.14; 4.01			3.13*	1.42; 4.83
Quintile monthly family income (vs Poorest)						
Poor			1.16	−0.28; 2.60		
Rich	−1.93	−4.02; 0.17			−1.73	−3.61; 0.15
Richest	−3.46*	−5.84; −1.09			−2.19*	−4.22; −0.17
Smoke (Yes vs no)	−1.40	−3.44; 0.63	−1.35	−2.85; 0.14		
Concurrent drug use (Yes vs no)			2.07	−0.45; 4.58		
Ever injected drugs (Yes vs no)					1.18	−0.35; 2.72
MMT model (Public facility vs private facility)	2.98*	1.24; 4.72	2.04*	0.85; 3.23	2.09*	0.62; 3.57
Quality of life during receiving MMT treatment (Unchanged vs better)	4.85*	1.04; 8.66			3.12	−0.46; 6.70
Supportive person in MMT (Yes vs no)						
None	−2.03	−4.18; 0.13				
Health workers at MMT facility	−1.45	−3.26; 0.37	−2.00*	−3.23; −0.77	−2.39*	−3.92; − 0.87
Neighbors/other acquaintances			2.51	−0.48; 5.51		
HIV test results (vs Negative)						
Unknown					2.25	−0.84; 5.35

* *p* < 0.05

## Discussion

This study presents empirical evidence for the psychological issues among patients receiving methadone maintenance treatment in different types of settings in Vietnam. The higher percentage of psychological problems including depression, anxiety, and stress was found among participants receiving treatment in public health facilities compared to those in private health facilities. In addition, predictors of mental health problems which based on multivariate regression model presented possible methods for identifying and preventing MMT patients who are at high risk of suffering from those psychological problems. The results highlight the need for implementing further mental healthcare services for MMT patients.

In this study, the rate of anxiety was the highest among psychological problems, which is consistent with the previous research [9]. In addition, more than two-thirds of opioid-dependent adults may undergo anxiety disorder during their lifetime [28]. The percentages of anxiety and depression in our study are lower than these rates in previous studies conducted among MMT patients in the rural parts and mountainous, remote areas of Vietnam [6, 22]. The difference can be explained by the fact that patients who live in mountainous areas have poorer access to healthcare services due to the lack of transportation, inadequate infrastructure, and high travel expense [29]. Moreover, a higher rate of experiencing stigma among MMT patients living in mountainous settings may contribute to the higher prevalence of psychological distress among those subjects [30]. The percentage of having psychological problems among MMT patients in other countries including the United States of America and Indonesia is higher than our finding, which may be due to the difference of culture [31–33]. The previous study also revealed that the lifetime rate of psychological disorders of Asia nations was lower than that of Western countries [34].

Interestingly, participants receiving MMT services in public health facilities had higher scores of DASS-21 in all measured psychological dimensions, compared to those having treatment in private health facilities. The poor performance of public sector services may result in the demand for generating private health facilities and private sectors account for 60% of outpatient contacts in Vietnam [16]. In term of accessibility, patients attending in private hospital tended to have shorter waiting time and reported better hospitality from providers compared to public facilities [20, 21]. Regarding responsiveness, the overwhelming workload may reduce the quality of public health facilities [35], for example, inadequate mental health counseling sessions [36] or lack of diagnosis explanation, especially which related to psychological problems [37]. In addition, stigmatization, a factor that can put MMT patients at higher risk of mental health issues and non-adherence to treatment [22], is also more common in public health facilities [38].

Our study also found that MMT patients with prolonged treatment duration greater than 9 years had lower scores in all psychological aspects, suggesting a lower risk of suffering from mental health problems. This situation can be explained by the fact that at the initial of receiving MMT, patients have to adapt to strict adherence [39] and face with securing their financial and social wellbeing during treatment duration [40, 41]. In this study, being unemployed and at poorer quintile groups also put MMT patients at higher risk of suffering from mental health problems. Moreover, long-term methadone patients, especially who involved in therapy continuously over many years, understand very well their treatment program with valuable insights on achieving reasonable goals and expectations [42]. Therefore, they may become more complacent because of either their physical health or mental health status recovered [39].

Several implications can be drawn from this study. First, screening for identifying psychological problems should be conducted in MMT clinics, especially in public health facilities. Patients found to suffer from mental health problems should take psychological counseling adequately. Second, taking account of the above discussions, facilitating employment and financial support will contribute to reducing the risk of undergoing psychological problems and should be implemented as a part of the primary care for MMT patients in Vietnam. Third, the reduction in DASS-21 observed in prolonged MMT duration suggests that more psychological assessment should be prioritized in the early stage of treatment. Integrating both physical and psychological care into MMT clinics and enhancing further related services to primary healthcare should be taken into account, especially when patients are at the initial period of the MMT program.

The strength of this study is using a validated international instrument (DASS-21) to assess psychological issues among MMT patients, which helps to improve the comparability between other studies worldwide and this study. However, several limitations should be acknowledged. First, a cross-sectional study design may limit the ability for establishing causal relationships among variables. Second, convenience sampling techniques and small sample size may decrease the capacity of generalizing the findings to the whole MMT patient population as well as reducing statistical power. Lastly, the information obtained by self-reported questionnaires may lead to recall bias and be influenced by social desirability.

## Conclusions

This study suggests that MMT patients receiving treatment in public health facilities might have a higher rate of psychological problems including depression, anxiety, and stress than that of those in the private health facility. Multiple factors predicting MMT patients who were at high risk of suffering from those psychological problems were also found. These results highlight the necessity of taking psychological counseling adequately for MMT patients and psychological assessment should be prioritized in the early stage of treatment.

## Data Availability

The data that support the findings of this study are available from the Institutional Review Board of National Institute of Hygiene and Epidemiology but restrictions apply to the availability of these data, which were used under license for the current study, and so are not publicly available. Data are however available from the authors upon reasonable request and with permission of from the Institutional Review Board of National Institute of Hygiene and Epidemiology.

## Abbreviations

MMT: Methadone Maintenance Treatment
VAS: Visual analog scale
